# Supplementary material for: The interactome of intact mitochondria by cross-linking mass spectrometry provides evidence for coexisting respiratory supercomplexes
Source: Mol Cell Proteomics. 2017 Dec 8;17(2):216–32. doi: 10.1074/mcp.RA117.000470 (PMC5795388; doi:10.1074/mcp.RA117.000470)
Supplement: Supplemental Data [file supp_RA117.000470_133922_0_supp_23978_fzffwf.zip › spectra_annotation/mito_spectra_annotation/331-1-8-1-6-1.pdf]

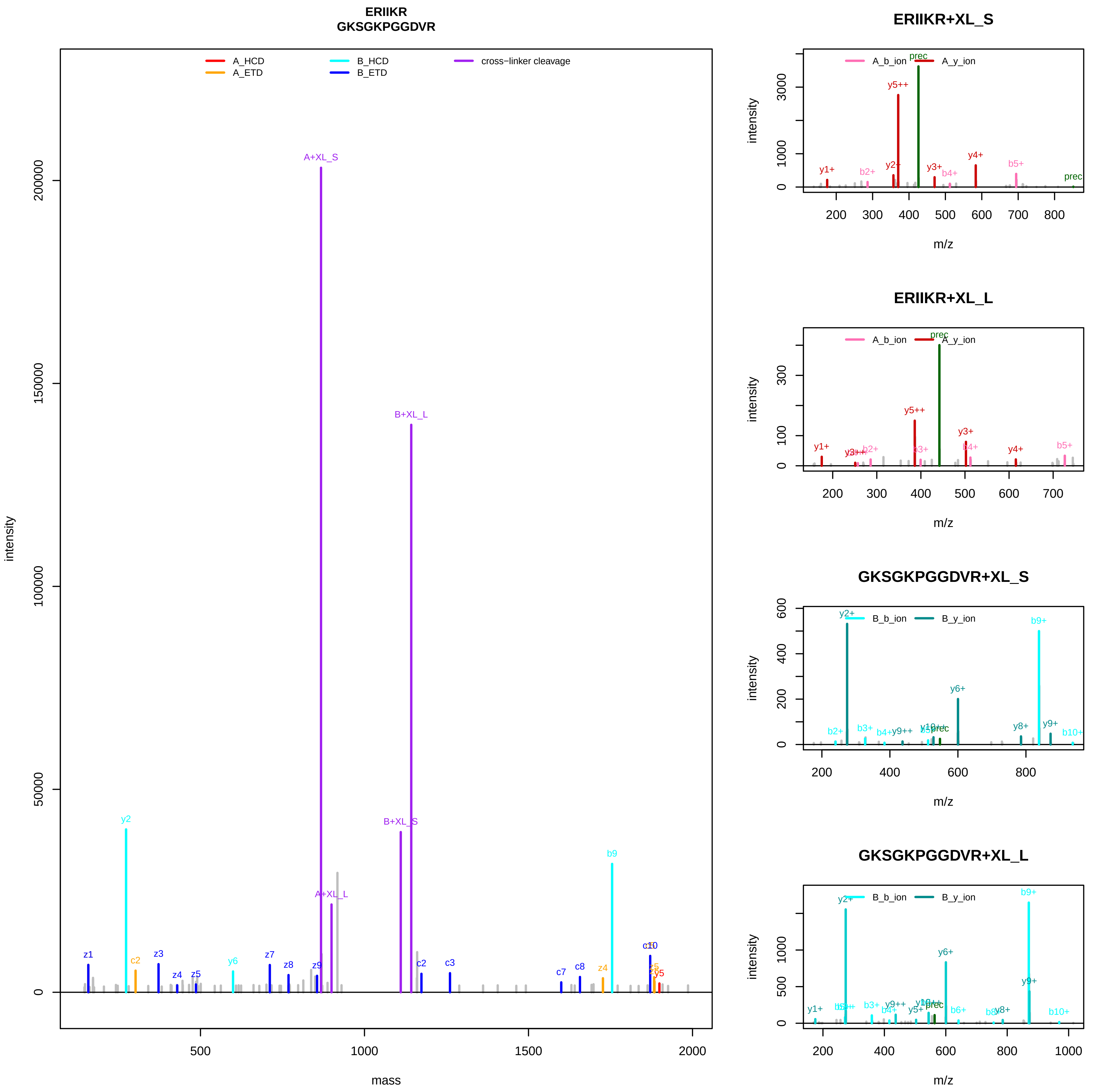
ERIIKR
GKSGKPGGDVR
A_HCD
A_ETD
B_HCD
B_ETD
cross−linker cleavage
A+XL_S
B+XL_L
B+XL_S
A+XL_L
z1
y2
c2
z3
z4
z5
y6
z7
z8
z9
c2
c3
c7
c8
z4
b9
c10
z5
y5
intensity
mass
0
50000
100000
150000
200000
500
1000
1500
2000
ERIIKR+XL_S
A_b_ion
A_y_ion
prec
y5++
y1+
b2+
y2+
y3+
b4+
y4+
b5+
ERIIKR+XL_L
y3++
b3+
y3+
b4+
y4+
b5+
GKSGKPGGDVR+XL_S
B_b_ion
B_y_ion
y2+
b9+
y6+
b2+
b3+
b4+
y9++
y8+
y9+
b10+
GKSGKPGGDVR+XL_L
y1+
b3+
y9++
y5+
b4+
y6+
b6+
y8+
b10+
m/z
intensity
